# Policy content and stakeholder network analysis for infant and young child feeding in Bangladesh

**DOI:** 10.1186/s12889-017-4338-0

**Published:** 2017-06-13

**Authors:** Sabrina Rasheed, Swapan Kumar Roy, Susmita Das, Syeda Nafisa Chowdhury, Mohammad Iqbal, Syeda Mahsina Akter, Khurshid Jahan, Shahadat Uddin, Anne Marie Thow

**Affiliations:** 1 Health Systems and Population Studies Division, International Centre for Diarrhoeal Disease Research Bangladesh (ICDDR,B), Dhaka, Bangladesh; 2 Bangladesh Breastfeeding Foundation, Dhaka, Bangladesh; 3 Complex Systems Research Group, The University of Sydney, Sydney, Australia; 4 Menzies Centre for Health Policy, Sydney School of Public Health, The University of Sydney, Sydney, Australia

**Keywords:** Content analysis, Stakeholder, IYCF, Policy

## Abstract

**Background:**

Appropriate infant and young child feeding (IYCF) practices are essential for nutrition of infants and young children. Bangladesh has one of the highest levels of malnutrition globally along with sub-optimal IYCF practices. A supportive policy environment is essential to ensure that effective IYCF interventions are scaled up.

The objectives of our study were to assess the support for IYCF in the national policy environment through policy analysis and stakeholder analysis and in so doing identify opportunities to strengthen the policy environment.

**Methods:**

We used a matrix developed by SAIFRN (the South Asian Infant Feeding Research Network) to systematically identify supportive national policies, plans and guidelines for IYCF. We adapted narrative synthesis and descriptive approaches to analyze policy content, based on four themes with a focus on support for mothers. We conducted three Net-Map interviews to identify stakeholders who influenced the policies and programs related to IYCF.

**Results:**

We identified 19 national policy documents relevant to IYCF. Overall, there was good level of support for IYCF practices at policy level – particularly regarding general support for IYCF and provision of information to mothers – but these were not consistently supported at implementation level, particularly regarding specificity and population coverage. We identified gaps regarding the training of health workers, capacity building, the monitoring and targeting of vulnerable mothers and providing an enabling environment to mothers, specifically with respect to maternity leave for working women. Urban populations and providers outside the public sector remained uncovered by policy. Our stakeholder analysis identified government entities such as the National Nutrition Service, as the most influential in terms of both technical and funding support as they had the mandate for formulation and implementation of policies and national programs. Stakeholders from different sectors played important roles, demonstrating the salience of IYCF.

**Conclusions:**

Although there is strong supportive policy environment for IYCF, it is important that policies cover all populations. Our analysis indicated that opportunities to strengthen the policy environment include: expanding population coverage, increasing inter-sector coordination, improving translation of policy objectives to implementation-level documents, and the engagement of non-public sectors. In addition, we recommend explicit strategies to engage diverse stakeholders in the formulation and implementation of IYCF policies.

**Electronic supplementary material:**

The online version of this article (doi:10.1186/s12889-017-4338-0) contains supplementary material, which is available to authorized users.

## Background

The potential effect of improving infant and young child feeding (IYCF) practices on reducing child malnutrition in Bangladesh is enormous [1, 2]. In poorer populations, malnutrition (stunting and wasting) rates of up to 49% and 17% respectively, have been reported among children less than five years of age in 2014 (BDHS), down slightly from 54% and 17.5% reported in the 2011 [3, 4]. Despite varying levels of undernutrition found across geographical and economic strata it is important to note that even household wealth does not eliminate malnutrition in Bangladesh - among the children under 5 residing in wealthiest households, 26% were stunted and 12% were wasted [4]. One of the main mediating behaviours that result in poor nutrition in early childhood is poor feeding practices [5]. In Bangladesh only 23% of 6–23 month old infants were fed according to recommended IYCF practices in 2014 [4]. Although the proportion of children for whom breastfeeding was initiated within an hour of birth, has increased from 47% (2011) to 51% (2014), the proportion of exclusive breastfeeding among infants under 6 months of age decreased during the same period (64% vs 55%) [3, 4]. Among children over 6 months of age, 25% consumed food with the minimum acceptable dietary diversity; and 21% of children consumed minimum acceptable diet [3].

Recommended IYCF practices reduce mortality and risks of non-communicable diseases while improving intelligence quotient (IQ), contributing directly to the achievement of Millennium Development Goals [6, 7] and Sustainable Development Goals [4]. Best practice interventions to support good IYCF practices are well established. The promotion of breastfeeding is found to be significantly cost effective [8] and improving complementary feeding is the third best investment among preventive interventions [9]. However, to enable the scaling up of interventions at a national scale, a supportive policy environment is essential [10].

Bangladesh has a long history of pro-active policy support for IYCF, and particularly breastfeeding. In 1981, the World Health Assembly passed the resolution on the International Code of Marketing of Breast Milk Substitutes and the government of Bangladesh introduced similar legislation in 1984 [11], which was further revised in 2013 [12]. At this time, a group of child health professionals recognized a decrease in breastfeeding rates and its effect on child health, and the Campaign for the Promotion and Protection of Breastfeeding was formalized in 1989 [11, 13]. In 1991, the Dhaka Declaration, a pledge to protect, promote and support breastfeeding, was signed by the President, the Prime Minister, Cabinet Ministers and participants following the International Innocenti Declaration of 1990 [11]. In 1994, the Director General of Health Services (DGHS) approved and initiated the implementation of a new hospital breastfeeding policy that included the 10 steps of the Baby-friendly Hospital Initiative. Between 1994 and 2009, the majority of hospitals (499 out of 650) in Bangladesh were certified as Baby Friendly [14]. In 2003 the global strategy for infant and young child feeding also created further impetus for development of national policies in Bangladesh and other countries around IYCF [15].

However, Bangladesh has considerable room for improvement in its policy environment for IYCF, based on a 2012 analysis using the World Breastfeeding Trend Initiative (WBTi) tool [16]. Building on WBTi analysis, and relevant policy theories regarding the importance of both policy content and stakeholders in policy making [17], the aim of this present study is to identify opportunities to strengthen the IYCF policy landscape in Bangladesh. We analyzed policy documents (including strategies, guidelines and other relevant documents) to assess gaps in the policies. We also conducted stakeholder analysis to obtain important insights about engaging with the stakeholders in future efforts to strengthen IYCF policies and programs.

## Methods

### Policy mapping

We identified potential areas of policy support for IYCF systematically, to delineate 1) relevant policy documents that could incorporate IYCF, and 2) relevant types of policy intervention, ranging from ‘high-level’ policies that provide strategic guidance for policy making (indicating political interest/will), sector-specific policies (such as health sector policies) and more focused implementation-relevant strategies and guidelines. As breastfeeding was a part of IYCF all policies that mentions breastfeeding was also included in the search. For policy analysis a matrix was developed with four domains of support for IYCF, in terms of:

a) Provision of high-level political support;

b) Provision of correct information to mothers;

c) Support training of health workers; and.

d) Enable mothers to engage with health care workers.

Documents were identified by searching the websites of the Ministry of Food and Disaster Management, Ministry of Women and Children Affairs, Ministry of Health and Family Welfare, Ministry of Labour and government archives. Documents were also identified through requests to Ministry of Health officials, and those from related Ministries, as well as from previous work. In order to achieve comprehensiveness, we cross-checked the list of documents with national level experts in IYCF. We identified the policies in existence between 2012 and 2013. We manually assessed the content of policies based on this matrix and used a narrative synthesis approach to present findings.

### Stakeholder analysis

The Net-Map method is a participatory interview technique that combines social network analysis [18], actor mapping [19] and power mapping [20]. It is an interview technique that examines the relative power, goals and perspectives of various stakeholders or actors, and looks at how they interact with each other. At the beginning of the Net-Map exercise, a list of potential stakeholders was identified based on their influence on policy and programs related to IYCF. Each group ranked the potential respondents of the preliminary list independently again based on above criteria. Interviewees were recruited from a range of relevant organizations through formal letters, emails and phone calls. For the Net-Map exercise, a total of 3 group interviews were conducted with at least 2 representatives from 5 groups of actors: 1) Government, 2) Research and academia, 3) Donor community, 4) Non-Government Organizations (NGOs) and 5) Individuals.

The social network analysis (SNA) data shows the connections and exchanges (referred to as links) between actors in the policy landscape. In our study, we decided to explore 2 specific types of links: funding and technical information. Interviewees were also asked to name actors (individual or organizations) who played an important role in the formulation of policies and programs in Bangladesh. They were then asked to attribute a power score, with no support as a score of zero and 5 as the highest level of support, to each actor named, depicting the level of influence that the underlying actor has over determining national policy and practice on IYCF. The participants were then asked to link the actors based on whether they provided or received technical support (for the technical support map). Interviewees were prompted collectively to only draw links that relate to IYCF issues. Actors in the network and links that connect them are reported here as stated by respondents rather than as they are supposed to be under any known formal systems. Hence, the maps are representative of the subjective views and experiences of respondents as key actors within those networks themselves.

The data scores provided by the Net-Map exercise was entered into excel sheets and analyzed using the software ORA (Organizational Risk Analyzer, copyright Carley, Carnegie Mellon University version). In this paper we used four network centrality measures to assess the actors and their linkages: in degree, out degree, betweenness and closeness centrality:

#### In-degree

This measure quantifies the tendency of an actor to receive input from the other network actors [21]. In other words, in-degree is a measure of receptivity or popularity.

#### Out-degree

This measure quantifies the tendency of an actor to provide input in terms of forming links with other network actors [21]. In other words, out-degree is a measure of expansiveness or activity of an actor in a network.

#### Betweenness

This centrality measure represents the capacity of an actor to control the flow of information between any pair of all other member actors in a network [21]. The underlying assumption of the betweenness centrality is that ‘actors in the middle’ have more ‘interpersonal influence’ on the others in a network [22].

#### Closeness

Closeness centrality represents the reachability of an actor from the other actors in a network [21]. An actor having high closeness centrality is well connected with the other network actors and vice versa.

The study was conducted during 2013–14. Approval for the study was taken from icddr,b Ethical Review Committee.

## Results and discussion

### Policy content analysis

A total of nineteen national policy documents were identified, as of December 2013, across 4 sectors: health, food and agriculture, labour, child development. General support for IYCF best practices are addressed in 14 documents; 11 documents specifically mention the provision of correct information to mothers through either behavior change communication or through regulation of information available; 8 documents addressed training of health workers; and 7 documents specify mention creating enabling conditions for mothers such as maternity leave and crèche *.*

### General support for infant and young child feeding

Among the high-level policy documents, the Prime Minister’s Declaration 2009 [23] and the Sixth Five Year Plan (2011–2015) [24] provide clear, general support for IYCF (Additional file 1) While the former focuses on providing an enabling environment in the workplace, along with general support for IYCF, the later promotes good nutritional practices, including specifying the benefits of exclusive breastfeeding for 6 months and continued breastfeeding up to 2 years; introduction of complementary foods of adequate nutritional quality and quantity after the age of 6 months; and improved hygiene practices including hand washing. These broad goals are supported in more specific sectoral policies of both health and non-health sectors, as detailed below.

Health sector specific strategies such as HPNSDP (2011–2016) [25] and the National Strategy for IYCF in Bangladesh (2007) [11] provide support for IYCF and detailed strategic direction regarding IYCF activities, stewardship, capacity building, communication and advocacy. The strategy specifically mentions development and distribution of specific guidelines for IYCF during emergency situations as well as for those affected by HIV. National Neonatal Health Strategy and Guidelines for Bangladesh (2009) supports optimum IYCF practices as a critical area for neonatal wellbeing and mentions it as part of Essential Newborn Care (ENC), emphasizing on training of field staff for supporting mothers in post-natal period [26].

As a cross cutting theme, operation plans of maternal, child and reproductive health and community-based health care support IYCF and promotion of breastfeeding activities [27, 28].

Moving towards implementation level policies, the National Food Policy Plan of Action (2008–2015) [29] and the National Plan of Action for Children (2004–2009) [30] supports promotion and protection of breastfeeding and complementary feeding. These include campaigns to improve breastfeeding and complementary feeding practices, implementation and monitoring of the Baby-Friendly Hospital Initiative, recommendations to increase maternity leave (discussed further in the section Enabling mothers / caregivers to engage with best practice interventions), enforcing Breast Milk Substitutes (BMS) Code and capacity building of frontline public health workers regarding IYCF for promoting optimum IYCF practices. Comprehensive Early Childhood Care and Development (ECCD) Policy Framework 2009 [31] mentions IYCF as a critical care practice in reference to early childhood development.

Although many of the policy documents from different ministries supported IYCF, the Ministry of Health and Family Welfare (MoHFW) remained the main ministry to implement the IYCF programs through the health systems using the existing health staff. Within the MoHFW, the National Nutrition Services (NNS) is the key agency assigned to oversee the policy and programs related to IYCF [32]. The NNS are mandated to mainstream nutrition, including IYCF, as part of the Strategic Plan for Health, Population and Nutrition Sector Development Program (2011–16).

### Provision of correct information to mothers / caregivers

In Bangladesh, provision of correct information to mothers and caregivers occurs in five broad ways: health service provider interaction, specific IYCF counseling, media campaigns, school/education-based approaches, and protection from information provided by industry (Additional file 1). The school-based approaches are meant to inform future generations through schools, rather than directly providing information to mothers.

Among the sector level strategy documents the National Food Policy Plan of Action (2008–2015) [29], National Plan of Action for Children Bangladesh (2004–2009) [30], and the National Neonatal Health Strategy and Guidelines for Bangladesh (2009) [26] identify the need to provide information to mothers through counseling, behaviour change communication and development of comprehensive educational material on breastfeeding and complementary feeding practices for the community. Specific support for IYCF counseling appears in the National IYCF Strategy (2007) as skilled behavior change counseling at all points of contact between mothers and health service providers both in facility and during community outreach [11]. This document also acknowledges that many NGOs are running similar maternal and child health programs and hence has similar contact points which can be used for providing IYCF support and reiterates the importance of uniformity of IYCF related information in all programs. The strategy emphasized the need for effectively counseling HIV-positive parents and other household members so that they can make informed infant feeding choices and are supported in carrying out their choice. Operational Plan for NNS (2011–2016) gives details of responsibilities for counseling activities at health facilities [32]. Although researchers have shown that scaling up well designed IYCF programs could improve IYCF practices in Bangladesh, it is important to note that the same research showed some very relevant limitation for effective programme implementation such as rapid transition of staff in key positions of implementing agencies, in government leadership, donors and other stakeholders [33].

The Breast Milk Substitute (BMS) (Regulation of Marketing) Act is intended to protect the mother from commercial influences on their infant feeding choices and to ensure that correct information on both breastfeeding and BMS reach the public [12]. The act also specifies that BMS cannot be donated or distributed, among the organizations or rescue shelters that are engaged in saving or reducing risk of death among children below five years of age or pregnant woman or newly delivered woman who are affected or endangered by natural calamity [12]. National Communication Framework and Plan for IYCF is another sector specific document that provides detailed communication strategy (BCC, communication for social change and advocacy) for IYCF messages [34]. Media campaigns, community dissemination, and counseling are some of the action plans that aim at providing information. The National Communication Framework and Plan for IYCF’s communication objectives include incorporation of IYCF in the National Curriculum in an effort to educate future generations.

There is a clear link between sectoral policy support for provision of IYCF information and implementation level documents for the HPNSDP (2011–2016), which mentions the Operational Plan for NNS (2011–2016) as the main implementation document. As IYCF is a cross-cutting theme involving multiple operational plans within MoHFW, Community Based Health Care also mention that IYCF will be integrated within their scope of service delivery through community clinics, the lowest level of public health facilities [27]. However, it is unclear how the effective implementation of the policy will be assessed over time.

### Training of frontline workers in IYCF

Among the high-level policies, the sixth Five Year Plan 2011–2015 [24] stated that the public sector health care workers should be trained to deliver IYCF services. Training of frontline workers in IYCF related skills is also mentioned in sectoral and implementation level policy documents of the health ministry, as a strategy to implement high-level policies. Among the sectoral policies Health, Population and Nutrition Sector Development Program 2011–2016 [25] and National Strategy for Infant and Young Child Feeding in Bangladesh 2007 [11] both mention the need for institutional and human capacity building for IYCF and other nutrition service delivery through the health sector. However, the National Communication Framework and Plan for IYCF [34] mentioned the need to train non-public sector workers in addition to the public sector workers. National Neonatal Health Strategy and Guidelines for Bangladesh 2009 [26] specifies training the health sector workers in terms of IYCF for neonatal period. Among the policy documents outside health sector National Plan of Action for Children Bangladesh 2004–2009 [30] mentions the need for training public health workers for IYCF. Researchers have reported the positive impact of training health care workers on child survival strategies such as IMCI on breastfeeding in Bangladesh [35].

Among the implementation documents, the Operational Plan for National Nutrition Services 2011–2016 [32] provides details about training the public sector workers on IYCF counseling including the schedule, duration and authority responsible for the training, this document also covers IYCF counseling, BFHI and BMS code [32]. Bangladesh National Training Module [36] then specifically describes the content of training for different health sector workers.

One gap that we observed is that although NGOs are mentioned as a key component of delivery of IYCF services, and thus doctors from NGOs are a priority stakeholder for training in the IYCF communication strategy [34], how they would be trained in IYCF is not mentioned. Also there is no specific mention of how the non-public sector workers will be trained on IYCF. In Bangladesh the MoHFW is responsible for primary and secondary health care services in rural area. In the urban area health service delivery is the responsibility of the Ministry of Local Government, Rural Development and Cooperatives and implemented by Local Government Division through city corporations and municipalities. In the policy documents, there is no mention of the training the urban health workers. In addition, how the quality of the training will be assessed or addressed is not mentioned in the policy documents.

### Enabling mothers / caregivers to engage with best practice interventions

Mothers and caregivers in Bangladesh are enabled to engage with best practice IYCF interventions through 1) supporting leave during the crucial phase of establishing breastfeeding and exclusive breastfeeding, 2) supporting mothers during emergency and 3) creation of supportive community networks.

Among the high-level policies, the National Children Policy 2011 [37] provides a general recommendation to “ensure the rights of safe birth and survival to all children” through extended maternity leave and provision of day care centres in the workplace. The document also mentions provision of breastfeeding corners during post-disaster period [37]. In line with this, Bangladesh Service Rules of 2012 [38] states that government servants can take six months of maternity leave. However, the policy specifies that one person is only eligible for maternity leave twice.

In the sectoral policies, National Food Policy Plan of Action 2008–2015 [29] recommends the provision of at least 5 months of maternity leave to working women and provision of day care centre at work places. The National Women Development Policy 2011 [39] focused on women’s development but mentions provision of 6 months of maternity leave, paternity leave and provision of an allowance to poor pregnant and lactating mothers under the social safety net for poverty alleviation. The policy recommends that laws are made to provide breastfeeding corners in industrial settings. National Labour Policy 2012 [40] provides general support for maternity leave for women working in factories but Bangladesh Labour Act 2006 (amended in 2013) [41] specifies the provision of 4 months of paid maternity leave for workers. The recommendation for maternity leave and the provision of crèches and breastfeeding corners is mentioned in several sectoral documents yet not followed through in the operations plan. The duration of maternity leave to be provided is also not uniform, ranging from 4 to 6 months in various documents. Further, there is no direction regarding the consequence of not incorporating maternity leave policy and how this may be enforced in practical terms. There are no policies to support provision of maternity leave in the private or informal sectors where many of the mothers are employed. In addition, there is no policy direction about ensuring maternity protection among women who work at management levels in different institutions.

Among the sectoral policy document National Strategy for Infant and Young Child Feeding in Bangladesh 2007 [11] articulates the need for community-based networks to help support appropriate IYCF at the community level, e.g. mother-to-mother support groups and peer or lay counselors (Additional file 1).The implementation-level documents mainly focused on supply side aspects of IYCF and there is no mention of strategies to create an enabling environment for the mothers or caregivers and therefore no budget for such a strategy exists.

### Stakeholder network analysis

For the network analysis, the final list of those who attended and the organisations they represented are provided in Table 1. As we aimed to get those who are knowledgeable about the policy and programs supporting IYCF, many of the individuals selected worked in different types of organizations beyond their current affiliation and some were powerful advocates of IYCF.

**Table 1:** Characteristics of the participants

Group	Number	Organizations represented
Government	7	DGHS, NNS, IPHN, MoHFW, Community Clinic project
UN/Donor	5	Food and Agriculture Organization, UNICEF, World Health organization, World Food program
NGO/Civil society	6	Save the children, World vision, Plan International, TAHN Foundation, Bangladesh Breastfeeding Foundation, BRAC
Research and Academia	7	icddr,b, International Food Policy Research Institute, Home Economics College, Medical college, Institute of Child and Mother Hospital, OGSB
Other	4	Kaler Kantho, Prothom Alo, The independent, Dr. Talukdar, Dr. Samina Chowdhury

### Stakeholders and their level of influence

The stakeholder network derived from Net-Map interviews identified 73 actors and is centralized around a handful of highly linked actors.

### Technical support (TS)

**Fig. 1:**
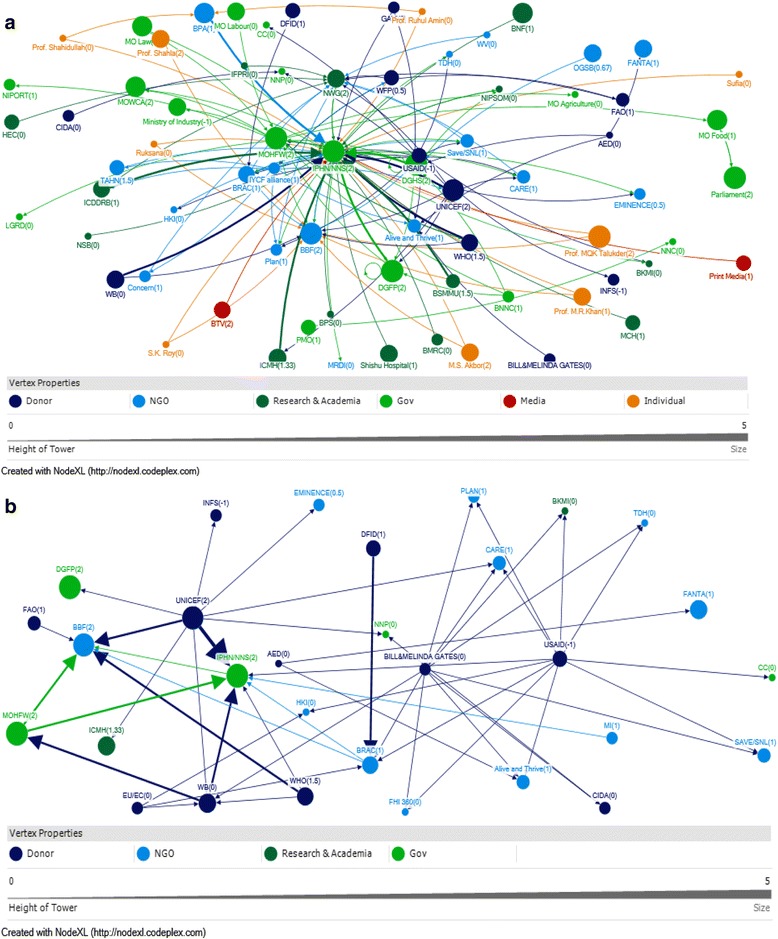
**a** Visual representation of technical support network among stakeholders and their attributes. **b** Visual representation of funding support network among stakeholders and their attributes

For technical support the network is specifically consolidated around the National Nutrition Services (NNS) within Institute of Public Health Nutrition (IPHN) (Fig. 1a). The NNS received the highest score for all the network measures indicating that it received a high-level of technical support (in-degree), provided technical support (out-degree), was accessible to other actors in the network (closeness centrality), and was influential over the flow of information between actors by its position in the network (betweenness centrality). The qualitative data revealed that NNS was the entity that was mandated by MoHFW to work on nutrition policy in Bangladesh and therefore, connected to other stakeholders in terms of IYCF program or policy.

In terms of receiving technical support (in-degree) the Nutrition Working Group (NWG), Bangladesh Breastfeeding Foundation (BBF) and Ministry of Health and Family Welfare (MoHFW) received the highest scores after the NNS. However, qualitative data revealed that any technical support that goes to NNS has to go through the MoHFW, despite this perception. NWG and BBF provided a platform for many organizations and individuals and therefore, were recipient of technical support. Among NGOs Brac, Alive and Thrive (A&T), CARE, and Plan were recipients of technical support (Table 2).

**Table 2:** Top ten entities based on network measures for technical support

Rank	In-degree	Out-degree	Betweenness	Closeness
1	NNS (36)	NNS (13)	NNS (3251.7)	NNS(0.010)
2	NWG (13)	MOHFW (10)	MOHFW (990.7)	UNICEF (0.007)
3	BBF (12)	UNICEF (10)	NWG (658.8)	MoHFW(0.007)
4	MOHFW (7)	IYCF alliance(10)	UNICEF (560.7	BBF (0.007)
5	DGHS (6)	USAID (10)	DGHS (419.3)	DGHS (0.006)
6	DGFP (5)	DGHS (5)	BBF (311.9)	NWG (0.006)
7	BRAC (4)	DGFP (3)	IYCF alliance(297.9)	IYCF alliance(0.006)
8	A &T (4)	BRAC (3)	USAID (267.0)	DGFP (0.006)
9	CARE (3)	WHO (3)	A&T (184.1)	USAID (0.006)
10	Plan (3)	WFP (3)	BRAC (163.2)	BRAC (0.006)

In terms of reaching to other actors (Out-degree) with technical support, public sector entities such as NNS, MoHFW, Director General Health Services (DGHS) and Director General Family Planning (DGFP) scored highly. UNICEF and IYCF alliance also scored highly. USAID and WHO also were within the top ten entities (Table 2).

In terms of being influential through mediating flows of technical support to others in the network (betweenness), again public sector entities such as NNS, MoHFW and DGHS received high scores. NWG, BBF and IYCF Alliance also were very influential. Among NGOs and projects A&T, BRAC received highest scores. UNICEF and USAID also came out as being influential in the network (Table 2).

In terms of ability to reach others in the network (closeness) again public sector entities received high scores. UNICEF was very close to others in the network. The three important networks that were able to easily reach others included BBF, NWG and IYCF alliance. Illustrating how these networks consist of many stakeholder organizations that are interested in nutrition and how they act as a hub for the exchange of ideas and information within the nutrition community. USAID and BRAC scored highly in terms of their ability to reach others (Table 2).

### Funding support

The funding network was mostly dominated by international donors, particularly USAID, Gates Foundation, UNICEF and European Union, which provided funding (out-degree); NNS, USAID,Gates foundation and the World Bank were accessible to a wide range of other actors in the network (closeness); and USAID, UNICEF, Gates Foudation and NNS exerted a high degree of control over the flow of funds between actors by their positions in the network (betweenness) (Fig. 1b; Table 3).

**Table 3:** Top ten entities based on network measures for funding support

b	In-degree	Out-degree	Betweenness	Closeness
1	NNS (7)	USAID (11)	USAID (267.8)	NNS (0.018)
2	BBF (6)	GATES (10)	UNICEF (252.8)	USAID (0.018)
3	BRAC (4)	UNICEF (9)	GATES (203.6)	GATES (0.017)
4	WB (4)	EU/EC (3)	NNS (165.9)	WB (0.017)
5	A&T (3)	WHO (3)	BRAC (113.9)	BRAC (0.016)
6	CARE (3)	BRAC (2)	A&T (109.4)	UNICEF (0.015)
7	BKMI (2)	WB (2)	WB (93.8)	CARE (0.015)
8	FHI 360 (2)	MOHFW (2)	BBF (80.8)	BBF (0.015)
9	HKI (2)	AED (2)	CARE (62.4)	A&T (0.014)
10	NNP (2)	NNS (1)	AED (56)	EU/EC (0.013)

In terms of funding support NNS was the recipient of most links (in degree) followed by BBF and other NGOs and programs (Fig. 1b; Table 3). The World Bank was also included as a recipient of funding support for IYCF policies and programs; qualitative data revealed that the World Bank was the administrator of multi-donor funds and therefore, were recipient of funds themselves. According to the qualitative data, the World Bank used the multi-donor fund to support the NNS operations plan. Government entities such as the MoHFW and NNS also provided some funding support to the network members (Table 3).

In terms of influencing the funding flow (betweenness) USAID, UNICEF, Gates and WB played a major role. NNS and BBF also influenced funding support to network members. Others who influenced funding support were NGOs and programs.

In terms of being able to reach out to others in the funding support network (closeness), the NNS scored the highest, closely followed by donors and UN entities such as USAID, Gates, WB, and UNICEF. Among the networks, BBF had close links with other actors. Among the NGOs, BRAC and Care were close to network members (Table 3).

### Stakeholder linkages by communities

**Fig. 2:**
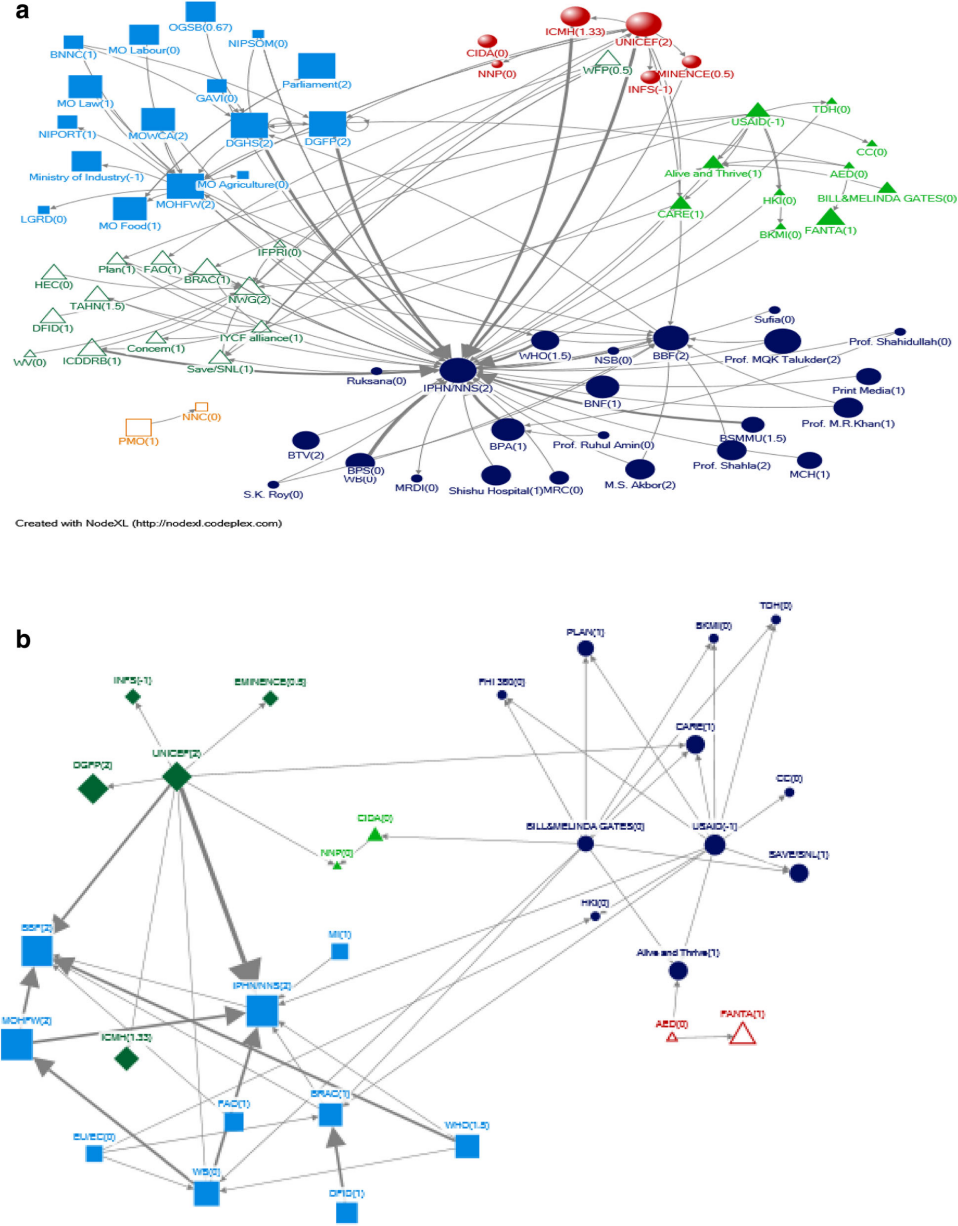
**a** Visual representation of community structure for technical support network. **b** Visual representation of community structure for funding support network

For technical support, stakeholders were aggregated onto 6 different communities based on clustering algorithms (Fig. 2a). The government actors form a distinct community and were linked to other communities through the Ministry of Health via NNS/IPHN. The second community consisted of UNICEF and a few academic and advocacy organizations. The third community consisted of donors and research and academic organizations. The fourth community centered on NNS and consisted of influential people as well as BBF, WHO, media and professional bodies. The fifth community with Nutrition Working Group in its center consisted of mostly networks, research and academia, Non-Government Organizations and a few donors. The final group shown had only two members, Prime Minister’s Office (PMO) and Bangladesh National Nutrition Council (BNNC). This community was not linked to other communities. Qualitative data showed that although the BNNC had a formal link with PMO with an understanding that this will provide the necessary prominence to the nutrition agenda, however the BNNC has become inactive over the years.

In the funding network, there were 3 clusters or communities (Fig. 2b). In one community UNICEF is the donor, and funds both public sector entities and research and academia. In the second community, the Gates Foundation and USAID are shown to be funding mostly NGOs. In the third community, the donors are the World Bank and WHO, and the major recipients are NNS and BBF.

## Discussion and conclusions

### Policy support for IYCF in Bangladesh

Bangladesh has history of formulating strong policies supporting International guidelines. The Prime Minister’s declaration shows support for IYCF at the highest level of Government. In the health sector policies, IYCF is supported from high-level policies to implementation documents, which are conducive to mainstreaming nutrition through the health systems especially in the rural areas. Other relevant Ministries also had policies that directly or indirectly support IYCF. From the stakeholder analysis we have seen that there are actors from many sectors actively engaged in IYCF policies and programs which show the importance of IYCF in multiple agendas. It is also important to note that National Nutrition Services are the core around which multiple actors direct their technical and funding support, which demonstrates that the government has taken a leadership role in this issue.

### Opportunities to strengthen the policy environment

Despite these strengths, we identified opportunities to strengthen the policy environment to better promote and protect IYCF with respect to maternity leave provisions, capacity development for IYCF counseling and multi-sectoral policy engagement. Although the best articulated policies supportive of IYCF were within the health sector, they partially or fully excluded large groups of women and children. In Bangladesh 57% women are in workforce [5]. For working women, maternity leave and crèche and breastfeeding corners in the workplace have been promised by the Prime Minister, these are yet to be implemented fully by the different Ministries. The existing policies are not aligned in terms of the length of maternity leave. In addition, the Ministry of Labour, the key Ministry concerned with working women, did not articulate the need for establishing crèches and breastfeeding corners at workplaces. These gaps in maternity protection affect almost 16.8 million working women in Bangladesh, many of who are employed in informal sector [42]. Further, the policies do not address women employed in the private sector. Another large group of people that the policy did not address was the 18.2 million people living in urban areas [5] whose primary health care needs are under the jurisdiction of the Ministry of Local Government Rural Development and Cooperatives [42]. This calls for a strengthening of the policies from different Ministries in terms of IYCF support, through engaging important stakeholders, and advocating for IYCF. Other researchers have shown that the different goals and interests of different parties, and their incorporation into policies from different sectors, in line with existing sectoral mandates, is one of the most difficult parts of the policy process in practice [43, 44], with nutrition, and therefore IYCF, often being seen as a secondary priority [45].

Support for IYCF needs to be translated from sectoral documents to operational documents in the non-health sector. We found that health is the only sector where IYCF was explicitly planned for in the implementation documents. In other sectors, although sectoral documents supported IYCF, few specific activities were formulated in this regard in the implementation documents. Given the availability powerful entities that provide technical support, such as MoHFW, NWG and UNICEF, it is important that more proactive implementation plans are formulated and advocated for in education, social protection, agriculture and labour sectors. Researchers have mentioned the importance of a national coordination mechanism, as mandated in the Global strategy, as a way to coordinates policies and programmes in other countries [46]. However, our participants did not mention any such process which would have been an important mechanism for incorporation of IYCF in policies and programs.

In the health sector policies the support for IYCF goes from high-level documents to implementation documents. The policy documents were specific about strategies to provide IYCF counseling through public health facilities. However, given the pluralistic nature of the health systems in Bangladesh [15], where both for-profit private sector and non-profit sectors provide health services, it is important to formulate strategies that engage these sectors in the implementation documents. Given that there are important stakeholders from diverse organizations who are involved in policy and programs of IYCF, it is important to engage them in formulating such strategies. Although our participants did not mention is, researchers have shown the impact of assessments of IYCF policies and programmes using the WBTi process on the policies and programmes in the country as they involved many important stakeholders [47] .

This research has used policy mapping and stakeholder analysis to improve understanding of the IYCF policy environment in Bangladesh, and to identify key opportunities to strengthen this aspect of IYCF intervention. Key strengths of the research approach are the in-depth consideration of policy content and actors, both influential in the policy environment [17], using a systematic approach. However, we also identified limitations to our research. Firstly, many high-level officials who were involved in the policy making had retired or moved on to other departments and were not able to participate in the meetings. Many of those who participated were not very close to the policy making process to talk about the actual dynamics. Second we did not analyze the institutional capacity to implement the policy. Third, our analysis was limited to policies and actors and did not look at the implementation process. Finally one of the important elements of policy development, namely, advocacy is missing in the manuscript. It will be useful to map organisations that are undertaking advocacy mainly with the government and other agencies in future studies and as evidence based advocacy is required to generate political will to development of policies and enact legislations.

### Lessons learned

Our analysis showed that there are substantial gaps in terms of population coverage, inter-sector coordination and engagement of non-public sectors in the policy documents that need to be addressed to strengthen support for IYCF. There are important and diverse stakeholders in the policy environment which clearly demonstrates the salience of IYCF in the agenda of different entities. However, there need to be explicit strategies to engage such stakeholders in the formulations and implementation of different policies that support IYCF.

## Additional file

Additional file 1: Summary of policies supporting best practice interventions. (PDF 72 kb)

## Abbreviations

A&T: Alive and Thrive
BBF: Bangladesh Breastfeeding Foundation
BCC: Behavior Change Communication
BDHS: Bangladesh Demographic and Health Survey
BFCI: Baby friendly community initiative
BFHI: Baby Friendly Hospital Initiative
BKMI: *Bangladesh Knowledge Management Initiative*
BNCC: Bangladesh National Nutrition Council
BRAC: Bangladesh Rural Advancement Committee
CARE: Cooperative for Assistance and Relief Everywhere
CHCP: Community Health Care Provider
DGFP: Directorate General of Family Planning
DGHS: Directorate General of Health Services
ECCD: Comprehensive Early Childhood Care and Development
EC: European Commission
ENC: Essential Newborn Care
EU: European Union
FHI: Family Health International
FWA: Family Welfare Assistant
FWV: Family Welfare Visitor
HA: Health Assistant
HI: Health Inspector
HKI: Helen Keller International
HIV/AIDS: Human Immuno Deficiency Virus/Acquired Immunodeficiency Syndrome
HPNSDP: Health Population and Nutrition Sector Development Program
IPHN: Institute of Public Health Nutrition
IPC: Interpersonal counseling
IYCF: Infant and young child feeding
IQ: Intelligence quotient
MCRAH: Maternal Child Reproductive and Adolescent Health
MOHFW: Ministry of Health and Family Welfare
NFP: National Food Policy
NNP: National Nutrition Program
NPAN: National Plan of Action for Nutrition
NNS: National Nutrition Services
NGO: Non-Government Organization
NWG: Nutrition Working Group
OP: Operations Plan
PMO: Prime Minister’s Office
PIP: Program Implementation Plan
RFW: Result framework
UHFWC: Union Health and Family Welfare Center
UHC: Upazila Health Complex
UNICEF: United Nations International Children’s Emergency Fund
USAID: United States Agency for International Development
WB: World Bank
WBTi: World Breastfeeding trend Initiative
WFP: World Food Programme
WHO: World Health Organization
