# Supplementary material for: Policy content and stakeholder network analysis for infant and young child feeding in Bangladesh
Source: BMC Public Health. 2017 Jun 13;17(Suppl 2):402. doi: 10.1186/s12889-017-4338-0 (PMC5496023; doi:10.1186/s12889-017-4338-0)
Supplement: Additional file 1: — Summary of policies supporting best practice interventions. (PDF 72 kb) [file 12889_2017_4338_MOESM1_ESM.pdf]

Additional file 1: Summary of policies supporting best practice interventions

**Policy**	**Description**	**Support for IYCF**				Details on IYCF in the policy
		General support for IYCF	Provision of correct information to mothers	Training of frontline workers on IYCF	Enable mothers / caregivers to engage with best practice interventions	
**High-level policies**						
1. Regulation of Marketing Act, 2013 [12]			x			This act is the regulatory measure to protect mothers, caregivers from breast milk substitutes (BMS) by a. Restrictions on promotion of BMS; b. Imposing restrictions on import, local production, sale of BMS; c. Mandating specific information relating to breast-milk substitutes being available; d. Making educational and other materials to promote breast milk available; and e. Provision of penalty for breach of the act.
2. Sixth Five Year Plan (SYFP)2011-2015 [24]	Ministry of Planning	x	x	x		This policy sets the target outcome level indicator for IYCF and gives *specific strategic* direction on IYCF practices. Broadly the strategies are implementation of National IYCF strategy; recommendation on stewardship for IYCF activities, training different cadre of public sector field staff in IYCF, linking of complementary feeding to multiple micronutrient program , social mobilization and BCC (behavior change communication) to promote specific IYCF practices , support and sustain IYCF through mainstreaming gender in nutrition programs.
3. Bangladesh Service Rules[38]	Public Service commission				x	Female government servants are entitled to six months of maternity leave with full pay. Permissibility of this benefit is restricted to two children in the previous version of the act.

4. National Children Policy2011[37]	Ministry of Women and Children Affairs				x	IYCF is not explicit in this policy document but provision of day care center by the employer for lactating and working mothers and 6 month maternity leave is mentioned. Provision of Breastfeeding Corner conducive for lactating mothers and newborn in post disaster state is mentioned.
5. Prime Minister's Declaration 2009[23]	Prime Minister's Office	x				Increasing maternity leave to 6 months, enabling environment for breastfeeding in all organization and public places (shopping centres etc.) and establishment of day care centre
**Sector specific policies**						
6. Health, Population and Nutrition Sector Development Program 2011-2016[25]	Ministry of Health and Family welfare	x	x	x		This health sector specific policy demarcates nutrition services under a separate operational plan- "National Nutrition Service (NNS)" for implementation through IPC (interpersonal counseling), BCC, BFHI (Baby-friendly Hospital Initiative) and BFCI (baby friendly community initiative), media campaign, breastfeeding week, high-level support for IYCF and BMS code, human resource and institutional capacity building for IYCF activities.
7. National Strategy for Infant and Young Child Feeding in Bangladesh 2007 [11]	Ministry of Health and Family welfare	x	x	x	x	It is a holistic IYCF specific strategy document and states 4 priority strategic areas for IYCF promotion in Bangladesh: 1. Legislation, policy, standards; 2.health system support, 3. Community-based support and 4. IYCF in exceptionally difficult circumstances.<br><br>The specific initiatives mentioned under these 4 areas are support implementation of the code of marketing of breast milk substitute, maternity protection in the workplace, standardized processed infant and complementary food, incorporation IYCF into national development

						policies and plans, ensure implementation of the BFHI, integrate IYCF support into all points of contact between mothers and health service providers , improve IYCF knowledge and skills of health service providers, develop community-based networks to help support appropriate IYCF at community level, develop capacity of the health system , community and families to support appropriate IYCF practices for children inflicted with HIV/AIDs, emergencies and malnutrition.
8. National Communication Framework and Plan for IYCF [34]	Ministry of Health and Family welfare	x	x	x		This communication framework is a component of the national IYCF strategy. It described three different approaches of communicating IYCF messages- advocacy, communication for social change and BCC. Action plan for implementation IYCF activities- training and orientation of government, NGO, private community workers, service providers and skilled birth attendants on including counseling, home visits, courtyard sessions, media campaign and advocacy.
9. National Food Policy Plan of Action2008-2015 **[29]**	Ministry of Food Disaster Management	x	x		x	The plan of action identifies promotion and protection of breastfeeding and improvement of complementary feeding practices as *key intervention* area to achieve NFP (National Food Policy) objective. Specific actions are ensuring safe and nutritious complementary feeding with local ingredients; strengthened BFHI; recommendation of at least 5 months maternity leave particularly post-partum; day care for working mothers; development of educational material for community; BMS Code compliance by the breast milk substitutes marketers through inter-ministerial efforts. It spells out the output indicators for the same, the time frame, responsible actors (both public and private) and

						verification means for the same programs.
10. National Plan of Action for Children Bangladesh 2004-2009 [30]	Ministry of Women and Children Affairs	x	x	x		Protection and promotion of breastfeeding and optimum IYCF practices through counseling and BCC, implementation of BFHI and its monitoring prioritizing hospitals with maternity services are mentioned in this policy document. Capacity building of health care staff in appropriate IYCF practices and provision of health services, and enforcement of law regarding breast milk substitute are also mentioned.
11. National Neonatal Health Strategy and Guidelines for Bangladesh2009 [26]	Ministry of Health and Family Welfare	x	x	x		IYCF is mentioned in reference to ENC (essential newborn care) and PNC (post-natal care). Optimum IYCF practices (including mothers with caesarian section), advocacy, institutional conduciveness, nutrition education, staff capacity building. The document also sets out IYCF outcome/output indicators, capacity building of health system staff to provide breastfeeding counseling to mothers during PNC visit.
12. Comprehensive Early Childhood Care and Development (ECCD) Policy Framework 2009 [31]	Ministry of Women and Children Affairs	x				Breastfeeding and complementary feeding are mentioned as critical care practices in reference to early child development. Guideline for caregivers mentions appropriate IYCF practices.
13. National Women Development Policy 2011 [39]	Ministry of Women and Children Affairs	x			x	Main focus of the policy was women's empowerment. The policy mentions provision of 6 months of maternity leave, paternity leave and provision of allowance to poor pregnant and lactating mothers under social safety net for poverty alleviation. The policy recommends that laws are made to provide breastfeeding corners in industrial settings. The policy also mentions need of providing correct information about maternal and child health to women is necessary however

						it does not mention specifically.
14. National Labour Policy 2012 [40]	Ministry of Labour				x	This policy provides general support for maternity leave for working women
15. Bangladesh Labour Act 2006 (amended in 2013) [41]	Ministry of law and parliamentary affairs				x	The focus of the act is provision of maternity leave for working women. In the 2006 version the policy mentions provision of 4 months of paid maternity leave.
**Implementation Documents**						
16. Operational Plan for National Nutrition Services2011-2016 [32]	Ministry of Health and Family Welfare	x	x	x		This document provides details of objectives, interventions, capacity building components, budgetary allocation for carrying out IYCF services and sets the indicators for monitoring and evaluation of IYCF services. IYCF counseling at Upazila: sub assistant community medical officer/ medical assistant and family welfare visitors deliver IYCF counseling (supervised by Medical Officer). IYCF counseling to all mothers with under 2 children visiting UHFWC(Union Health and Family Welfare Center), nutrition counseling to pregnant, lactating and adolescent girls. Details given on content.<br>IYCF counseling and advocacy at community clinic level by Health assistant, Family Welfare Assistant, and Community Health Care Providers. Capacity building schedule as described in HPNSDP (program manager, nurse, doctor, health worker). Training on BMS code for high-level policy makers, doctors, nurses and medical officer UHC. Capacity building regarding mainstreaming nutrition includes high-level policy makers and doctors from NGO sector also. It provides IYCF related relevant RFW (Result framework), PIP (Program

						Implementation Plan) and OP level indicators. Year wise budget for IYCF, BFHI, BMS code.
17. Operational Plan Community Based Health Care [27]	DGHS (July 2011 -June 2016)	x	x			IYCF will be one of the main activities of the community clinic and will be done through BCC and counseling and also gives objectively verifiable indicators.
18. Operational Plan Maternal, Child, Reproductive and Adolescent Health (MCRAH)[28]	Directorate General of Family Planning (DGFP)	x				As a part mainstreaming maternal and child nutrition in DGFP activities IYCF is also included in this operational plan (OP) as it is a cross cutting theme.
19. Bangladesh National Training Module [36]	Ministry of Health and Family Welfare 2011	x	x	x		Manual to train health care staff, program managers who support IYCF for improving mother's skill and information. Sessions like how to improve the performance of health workers, importance of breastfeeding, position, attachment and manual, expression of milk during breastfeeding, breastfeeding difficulties and how to address them, complementary feeding, counseling practices on breastfeeding and complementary feeding are aimed at improving the practical and counseling skills of the trainee.
